# Gallic acid pyridine monosolvate

**DOI:** 10.1107/S1600536811043868

**Published:** 2011-10-29

**Authors:** Fu-Yue Dong, Jie Wu, Hai-Yan Tian, Qing-Mei Ye, Ren-Wang Jiang

**Affiliations:** a Guangdong Province Key Laboratory of Pharmacodynamic Constituents of Traditional Chinese Medicine and New Drugs Research, Institute of Traditional Chinese Medicine and Natural Products, Jinan University, Guangzhou 510632, People’s Republic of China

## Abstract

In the title compound (systenatic name: 3,4,5-trihy­droxy­benzoic acid pyridine monosolvate), C_5_H_5_N·C_7_H_6_O_5_, the gallic acid mol­ecule is essentially planar (r.m.s deviation = 0.0766 Å for non-H atoms) and is linked to the pyridine mol­ecule by an O—H⋯N hydrogen bond. An intra­molecular O—H⋯O hydrogen bond occurs in the gallic acid mol­ecule. The gallic acid and pyridine mean planes make a dihedral angle 12.6 (3)°. Inter­molecular O—H⋯O and O—H⋯N hydrogen bonding involving the hy­droxy and carboxyl groups and the pyridine mol­ecule, and π–π inter­actions between inversion-related pyridines [centroid–centroid distance = 3.459 (6) Å] and between pyridine and benzene rings [centroid–centroid distance = 3.548 (6) Å], lead to a three-dimensional network in the crystal.

## Related literature

For the biological activity of gallic acid, see: Souza *et al.* (2011 ▶); Ozcelik *et al.* (2011 ▶); Liu *et al.* (2011 ▶). For previous reports on the crystal structures of gallic acid monohydrate and gallic acid monopyridine solvate, see: Clarke *et al.* (2011 ▶); Jiang *et al.* (2000 ▶). For π–π inter­actions in natural flavonoids, see: Jiang *et al.* (2002 ▶, 2009 ▶).
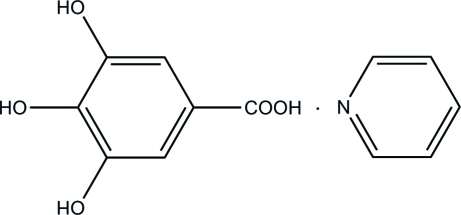


## Experimental

### 

#### Crystal data

C_5_H_5_N·C_7_H_6_O_5_
*M* *_r_* = 249.22
Monoclinic, 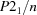

*a* = 9.335 (1) Å
*b* = 10.435 (2) Å
*c* = 11.8581 (15) Å
β = 107.632 (8)°
*V* = 1100.9 (3) Å^3^
*Z* = 4
Mo *K*α radiation
μ = 0.12 mm^−1^
*T* = 293 K
0.34 × 0.20 × 0.12 mm

#### Data collection

Bruker SMART CCD 1000 diffractometer
Absorption correction: multi-scan (*SADABS*; Sheldrick, 2004 ▶) *T*_min_ = 0.821, *T*_max_ = 0.986
2601 measured reflections
1944 independent reflections
1031 reflections with *I* > 2σ(*I*)
*R*_int_ = 0.057

#### Refinement

*R*[*F* ^2^ > 2σ(*F* ^2^)] = 0.066
*wR*(*F* ^2^) = 0.172
*S* = 1.02
1944 reflections
166 parameters
H-atom parameters constrained
Δρ_max_ = 0.36 e Å^−3^
Δρ_min_ = −0.30 e Å^−3^

### 

Data collection: *SMART* (Bruker, 1998 ▶); cell refinement: *SAINT* (Bruker, 1998 ▶); data reduction: *SAINT*; program(s) used to solve structure: *SHELXTL* (Sheldrick, 2008 ▶); program(s) used to refine structure: *SHELXTL*; molecular graphics: *XP* in *SHELXTL*; software used to prepare material for publication: *SHELXTL*.

## Supplementary Material

Crystal structure: contains datablock(s) I, global. DOI: 10.1107/S1600536811043868/pk2351sup1.cif

Structure factors: contains datablock(s) I. DOI: 10.1107/S1600536811043868/pk2351Isup2.hkl

Supplementary material file. DOI: 10.1107/S1600536811043868/pk2351Isup3.cml

Additional supplementary materials:  crystallographic information; 3D view; checkCIF report

## Figures and Tables

**Table 1:** Hydrogen-bond geometry (Å, °)

*D*—H⋯*A*	*D*—H	H⋯*A*	*D*⋯*A*	*D*—H⋯*A*
O1—H1*A*⋯O2^i^	0.82	2.12	2.869 (3)	152
O1—H1*A*⋯O2	0.82	2.34	2.736 (4)	110
O2—H2*A*⋯O5^ii^	0.82	1.87	2.675 (4)	166
O3—H3*A*⋯O4^iii^	0.82	1.91	2.718 (3)	169
O4—H4*A*⋯N1	0.82	1.92	2.730 (4)	169

Symmetry codes: (i) 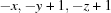; (ii) 
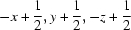
; (iii) 
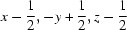
.

**Fig. 1.**
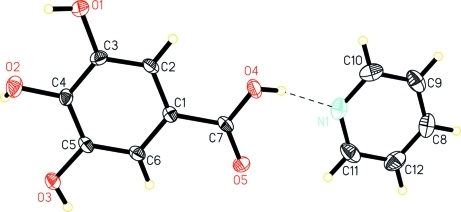
The molecular structure of the title compound showing 30% probability displacement ellipsoids.

**Fig. 2.**
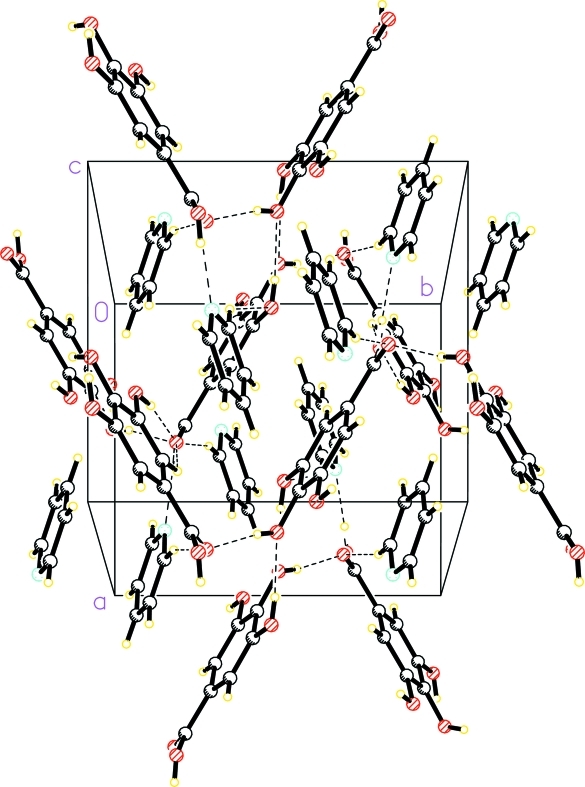
The packing diagram viewed approximately down the c-axis.

## supplementary crystallographic information

### Comment

Gallic acid, a dietary polyphenol, is widely distributed in many edible and medicinal plants. It can exist as a single molecule or as a structural unit of hydrolysable tannins. It has been found to show strong pharmacological activities including antioxidant (Souza, *et al.* 2011), antiviral (Ozcelik, *et al.*, 2011) and antitumor properties (Liu, *et al.*, 2011). This compound contains two of the most common functional groups in natural products, e.g. carboxylic acid and phenolic groups. Crystal engineering studies have revealed interesting polymorphism. Four polymorphs of the monohydrate of gallic acid with three space groups (P 2_1_/c, P 2/n, and P 1), and an anhydrous form with space group C 2/c have been reported (Clarke *et al.*, 2011).  We report herein the pyridine monosolvate of gallic acid.

The gallic acid molecule is essentially planar. The mean deviation of the benzene ring is 0.0030 Å, which is similar to that in gallic acid monohydrate (0.0028 Å), and its dihedral angle with the plane of the carboxyl group is 9.8 (3) °, which is larger than that in gallic acid monohydrate (2.9°) (Jiang, *et al.*, 2000).  The gallic acid and pyridine molecules make a dihedral angle of 12.8 (4) °.  The bond distances are all normal.

Within the asymmetric unit, the gallic acid molecule and pyridine molecule are linked through hydrogen bond O4–H···N1. Intermolecular O—H···O and O—H···N hydrogen-bonding interactions involving the hydroxyl and carboxylic acid groups and the pyridine molecule (Table 1) form a supramolecular assembly. A short intramolecular C—H···O interaction between the C10 methine and a hydroxyl O acceptor is also present [C10–H···O5, 3.169 (18) Å; <C–H···O, 162.0 (5) °]. It is noteworthy that π-π interactions play an important role in the molecular packing. The gallic acid molecules show π-π interactions with the pyridine molecules [centroid-centroid distance 3.548 (6) Å and displacement angle 12.8 (3) °], and inversion-related pyridine molecules are also linked by π-π interactions [centroid-centroid distance = 3.459 (6) Å]. The centroid-centroid distances observed in gallic acid monopyridine solvate are significantly shorter than those in natural flavonoids (Jiang, *et al.*, 2009 and 2002).

### Experimental

The title compound was extracted from the whole plant of Polygonum chinense L. The dried plant material (5 kg) was powdered and extracted with 95% ethanol at room temperature to afford the crude extract, which was suspended in distilled water and partitioned with petroleum ether, ethyl acetate and n-butanol. The n-butanol fraction (100g) was subjected to macroporous resin, reverse phase silica gel chromatography to give compound I (21 mg), which was recrystallized in pyridine to afford the monopyridine solvate of gallic acid.

### Refinement

The C-bound H atoms were positioned geometrically and were included in the refinement in the riding-model approximation, with C—H = 0.96 Å (CH_3_) and *U*_iso_(H) = 1.5*U*_eq_(C); 0.97 Å (CH_2_) and *U*_iso_(H) = 1.2*U*_eq_(C); 0.93 Å (aryl H) and *U*_iso_(H)= 1.2*U*_eq_(C); O—H = 0.82 Å and *U*_iso_(H) = 1.5*U*_eq_(O).

### Crystal data

**Table:**

C_5_H_5_N·C_7_H_6_O_5_	*F*(000) = 520
*M**_r_* = 249.22	*D*_x_ = 1.504 Mg m^−^^3^
Monoclinic, *P*2_1_/*n*	Mo *K*α radiation, λ = 0.71073 Å
Hall symbol: -P 2yn	Cell parameters from 2601 reflections
*a* = 9.335 (1) Å	θ = 2.5–25.0°
*b* = 10.435 (2) Å	µ = 0.12 mm^−^^1^
*c* = 11.8581 (15) Å	*T* = 293 K
β = 107.632 (8)°	Prism, colorless
*V* = 1100.9 (3) Å^3^	0.34 × 0.20 × 0.12 mm
*Z* = 4	

### Data collection

**Table:**

Bruker SMART CCD 1000 diffractometer	1944 independent reflections
Radiation source: fine-focus sealed tube	1031 reflections with *I* > 2σ(*I*)
graphite	*R*_int_ = 0.057
ω scan	θ_max_ = 25.0°, θ_min_ = 2.5°
Absorption correction: multi-scan (*SADABS*; Sheldrick, 2004)	*h* = −1→11
*T*_min_ = 0.821, *T*_max_ = 0.986	*k* = −1→12
2601 measured reflections	*l* = −14→13

### Refinement

**Table:**

Refinement on *F*^2^	Secondary atom site location: difference Fourier map
Least-squares matrix: full	Hydrogen site location: inferred from neighbouring sites
*R*[*F*^2^ > 2σ(*F*^2^)] = 0.066	H-atom parameters constrained
*wR*(*F*^2^) = 0.172	*w* = 1/[σ^2^(*F*_o_^2^) + (0.0724*P*)^2^] where *P* = (*F*_o_^2^ + 2*F*_c_^2^)/3
*S* = 1.02	(Δ/σ)_max_ < 0.001
1944 reflections	Δρ_max_ = 0.36 e Å^−^^3^
166 parameters	Δρ_min_ = −0.30 e Å^−^^3^
0 restraints	Extinction correction: *SHELXTL* (Sheldrick, 2008), Fc^*^=kFc[1+0.001xFc^2^λ^3^/sin(2θ)]^-1/4^
Primary atom site location: structure-invariant direct methods	Extinction coefficient: 0.026 (5)

### Special details

**Table:**

Geometry. All esds (except the esd in the dihedral angle between two l.s. planes) are estimated using the full covariance matrix. The cell esds are taken into account individually in the estimation of esds in distances, angles and torsion angles; correlations between esds in cell parameters are only used when they are defined by crystal symmetry. An approximate (isotropic) treatment of cell esds is used for estimating esds involving l.s. planes.
Refinement. Refinement of F^2^ against ALL reflections. The weighted R-factor wR and goodness of fit S are based on F^2^, conventional R-factors R are based on F, with F set to zero for negative F^2^. The threshold expression of F^2^ > 2sigma(F^2^) is used only for calculating R-factors(gt) etc. and is not relevant to the choice of reflections for refinement. R-factors based on F^2^ are statistically about twice as large as those based on F, and R- factors based on ALL data will be even larger.

### Fractional atomic coordinates and isotropic or equivalent isotropic displacement parameters (Å^2^)

**Table:**

	*x*	*y*	*z*	*U*_iso_*/*U*_eq_	
O1	0.2507 (3)	0.4817 (3)	0.5719 (2)	0.0455 (9)	
H1A	0.1613	0.4918	0.5650	0.068*	
O2	0.0218 (3)	0.5026 (3)	0.3644 (3)	0.0478 (9)	
H2A	−0.0003	0.5559	0.3111	0.09 (2)*	
O3	0.0523 (3)	0.3953 (3)	0.1596 (2)	0.0443 (9)	
H3A	0.0722	0.3559	0.1064	0.026 (12)*	
O4	0.6443 (3)	0.2100 (3)	0.4836 (2)	0.0447 (9)	
H4A	0.7301	0.2041	0.4794	0.067*	
O5	0.5775 (3)	0.1955 (3)	0.2878 (2)	0.0372 (8)	
C1	0.4139 (4)	0.3050 (4)	0.3759 (3)	0.0272 (10)	
C2	0.3957 (4)	0.3632 (4)	0.4761 (3)	0.0335 (10)	
H2B	0.4718	0.3581	0.5478	0.040*	
C3	0.2654 (4)	0.4285 (4)	0.4696 (3)	0.0319 (10)	
C4	0.1511 (4)	0.4397 (4)	0.3634 (3)	0.0293 (10)	
C5	0.1690 (4)	0.3824 (4)	0.2629 (3)	0.0299 (10)	
C6	0.2988 (4)	0.3151 (4)	0.2682 (3)	0.0308 (10)	
H6A	0.3096	0.2768	0.2004	0.037*	
C7	0.5536 (4)	0.2314 (4)	0.3809 (3)	0.0298 (10)	
N1	0.9126 (4)	0.1750 (4)	0.4402 (4)	0.0467 (10)	
C8	1.1809 (5)	0.0925 (5)	0.4259 (5)	0.0508 (13)	
H8A	1.2731	0.0630	0.4216	0.061*	
C9	1.1691 (5)	0.1366 (5)	0.5296 (5)	0.0544 (14)	
H9A	1.2532	0.1387	0.5962	0.065*	
C10	1.0337 (6)	0.1779 (5)	0.5367 (4)	0.0516 (14)	
H10A	1.0251	0.2082	0.6082	0.062*	
C11	0.9235 (6)	0.1326 (5)	0.3370 (4)	0.0529 (14)	
H11A	0.8389	0.1312	0.2708	0.063*	
C12	1.0599 (6)	0.0908 (5)	0.3285 (4)	0.0542 (14)	
H12A	1.0685	0.0619	0.2567	0.065*	

### Atomic displacement parameters (Å^2^)

**Table:**

	*U*^11^	*U*^22^	*U*^33^	*U*^12^	*U*^13^	*U*^23^
O1	0.0410 (17)	0.066 (2)	0.0299 (16)	0.0120 (17)	0.0106 (13)	−0.0052 (16)
O2	0.0428 (18)	0.065 (2)	0.0382 (17)	0.0205 (18)	0.0164 (14)	0.0152 (18)
O3	0.0319 (16)	0.067 (2)	0.0288 (16)	0.0128 (16)	0.0009 (13)	−0.0029 (17)
O4	0.0269 (15)	0.071 (2)	0.0322 (16)	0.0146 (17)	0.0036 (13)	0.0038 (16)
O5	0.0367 (16)	0.051 (2)	0.0277 (15)	0.0034 (15)	0.0150 (12)	−0.0043 (15)
C1	0.024 (2)	0.032 (2)	0.026 (2)	−0.0016 (19)	0.0081 (17)	0.0049 (18)
C2	0.027 (2)	0.044 (3)	0.024 (2)	−0.001 (2)	−0.0009 (17)	−0.002 (2)
C3	0.036 (2)	0.036 (3)	0.026 (2)	−0.001 (2)	0.0132 (19)	−0.0036 (19)
C4	0.027 (2)	0.035 (3)	0.026 (2)	0.009 (2)	0.0088 (17)	0.0079 (19)
C5	0.022 (2)	0.038 (3)	0.026 (2)	−0.003 (2)	0.0023 (17)	0.0054 (19)
C6	0.026 (2)	0.043 (3)	0.0227 (19)	−0.005 (2)	0.0056 (16)	0.0005 (19)
C7	0.0213 (19)	0.039 (3)	0.026 (2)	−0.005 (2)	0.0024 (17)	0.003 (2)
N1	0.036 (2)	0.043 (3)	0.065 (3)	0.0048 (19)	0.021 (2)	0.005 (2)
C8	0.042 (3)	0.039 (3)	0.078 (4)	0.006 (2)	0.028 (3)	0.013 (3)
C9	0.037 (3)	0.054 (3)	0.060 (3)	−0.008 (3)	−0.003 (2)	0.011 (3)
C10	0.067 (3)	0.048 (3)	0.047 (3)	−0.012 (3)	0.028 (3)	−0.009 (3)
C11	0.054 (3)	0.048 (3)	0.043 (3)	−0.003 (3)	−0.006 (2)	0.012 (3)
C12	0.077 (4)	0.049 (3)	0.049 (3)	0.004 (3)	0.038 (3)	0.002 (3)

### Geometric parameters (Å, °)

**Table:**

O1—C3	1.379 (5)	C4—C5	1.388 (5)
O1—H1A	0.8200	C5—C6	1.385 (5)
O2—C4	1.377 (5)	C6—H6A	0.9300
O2—H2A	0.8200	N1—C11	1.333 (6)
O3—C5	1.378 (4)	N1—C10	1.343 (6)
O3—H3A	0.8200	C8—C9	1.348 (7)
O4—C7	1.275 (4)	C8—C12	1.349 (7)
O4—H4A	0.8200	C8—H8A	0.9300
O5—C7	1.248 (4)	C9—C10	1.362 (7)
C1—C2	1.391 (5)	C9—H9A	0.9300
C1—C6	1.402 (5)	C10—H10A	0.9300
C1—C7	1.499 (5)	C11—C12	1.379 (7)
C2—C3	1.375 (5)	C11—H11A	0.9300
C2—H2B	0.9300	C12—H12A	0.9300
C3—C4	1.387 (5)		
			
C3—O1—H1A	109.5	C1—C6—H6A	120.1
C4—O2—H2A	109.5	O5—C7—O4	123.2 (4)
C5—O3—H3A	109.5	O5—C7—C1	120.3 (3)
C7—O4—H4A	109.5	O4—C7—C1	116.5 (3)
C2—C1—C6	119.2 (4)	C11—N1—C10	120.8 (4)
C2—C1—C7	121.3 (3)	C9—C8—C12	120.4 (5)
C6—C1—C7	119.5 (3)	C9—C8—H8A	119.8
C3—C2—C1	120.2 (3)	C12—C8—H8A	119.8
C3—C2—H2B	119.9	C8—C9—C10	119.9 (5)
C1—C2—H2B	119.9	C8—C9—H9A	120.1
C2—C3—O1	118.2 (3)	C10—C9—H9A	120.1
C2—C3—C4	121.1 (4)	N1—C10—C9	119.8 (4)
O1—C3—C4	120.7 (4)	N1—C10—H10A	120.1
O2—C4—C3	117.9 (4)	C9—C10—H10A	120.1
O2—C4—C5	123.1 (3)	N1—C11—C12	120.0 (5)
C3—C4—C5	118.9 (4)	N1—C11—H11A	120.0
O3—C5—C6	122.3 (4)	C12—C11—H11A	120.0
O3—C5—C4	116.9 (4)	C8—C12—C11	119.1 (5)
C6—C5—C4	120.7 (3)	C8—C12—H12A	120.5
C5—C6—C1	119.8 (4)	C11—C12—H12A	120.5
C5—C6—H6A	120.1		
			
C6—C1—C2—C3	0.9 (6)	C4—C5—C6—C1	−0.2 (6)
C7—C1—C2—C3	−179.0 (4)	C2—C1—C6—C5	−0.2 (6)
C1—C2—C3—O1	178.3 (4)	C7—C1—C6—C5	179.7 (4)
C1—C2—C3—C4	−1.2 (6)	C2—C1—C7—O5	−169.7 (4)
C2—C3—C4—O2	177.8 (4)	C6—C1—C7—O5	10.4 (6)
O1—C3—C4—O2	−1.6 (6)	C2—C1—C7—O4	9.5 (6)
C2—C3—C4—C5	0.8 (6)	C6—C1—C7—O4	−170.3 (4)
O1—C3—C4—C5	−178.7 (4)	C12—C8—C9—C10	1.0 (8)
O2—C4—C5—O3	2.7 (6)	C11—N1—C10—C9	−0.4 (7)
C3—C4—C5—O3	179.6 (4)	C8—C9—C10—N1	−0.2 (8)
O2—C4—C5—C6	−177.0 (4)	C10—N1—C11—C12	0.2 (7)
C3—C4—C5—C6	−0.1 (7)	C9—C8—C12—C11	−1.2 (8)
O3—C5—C6—C1	−179.9 (4)	N1—C11—C12—C8	0.6 (7)

### Hydrogen-bond geometry (Å, °)

**Table:**

*D*—H···*A*	*D*—H	H···*A*	*D*···*A*	*D*—H···*A*
O1—H1A···O2^i^	0.82	2.12	2.869 (3)	152.
O1—H1A···O2	0.82	2.34	2.736 (4)	110.
O2—H2A···O5^ii^	0.82	1.87	2.675 (4)	166.
O3—H3A···O4^iii^	0.82	1.91	2.718 (3)	169.
O4—H4A···N1	0.82	1.92	2.730 (4)	169.

Symmetry codes: (i) −*x*, −*y*+1, −*z*+1; (ii) −*x*+1/2, *y*+1/2, −*z*+1/2; (iii) *x*−1/2, −*y*+1/2, *z*−1/2.
